# Evaluation of intravenous voriconazole in patients with compromised renal function

**DOI:** 10.1186/1471-2334-13-14

**Published:** 2013-01-16

**Authors:** Craig M Lilly, Verna L Welch, Thomas Mayer, Paul Ranauro, Joanne Meisner, David R Luke

**Affiliations:** 1 University of Massachusetts Medical School, Worcester, Massachusetts, USA; 2 Medical Affairs, Pfizer Inc, Collegeville, Pennsylvania, USA

**Keywords:** Voriconazole, Caspofungin, Fluconazole, Renal dysfunction, Sulfobutylether-β-cyclodextrin, SBECD, Acute kidney injury

## Abstract

**Background:**

Incorporation of the solubilizing excipient, sulfobutylether-β-cyclodextrin (SBECD), in the intravenous (IV) formulation of voriconazole has resulted in the recommendation that this formulation be used with caution in patients with creatinine clearances (Cl_cr_) < 50 mL/min. This study evaluated the safety of IV voriconazole compared with two other IV antifungals not containing SBECD in patients with compromised renal function.

**Methods:**

A total of 128 patients aged 11–93 years who had a baseline Cl_cr_ < 50 mL/min between January 1, 2007 and December 31, 2010 were identified from a database of a university-affiliated inpatient healthcare system; of these, 55 patients received caspofungin, 54 patients received fluconazole, and 19 patients received voriconazole. Changes in serum creatinine (S_cr_) and Cl_cr_ levels while on therapy were compared with baseline values and between groups.

**Results:**

The groups had similar characteristics apart from the larger proportion of females that received fluconazole. Baseline S_cr_ was higher in those receiving caspofungin, but maximal increases of S_cr_ and decreases in Cl_cr_ were greatest for the fluconazole group. Acute kidney injury (AKI), assessed by RIFLE criteria, was more frequent in the fluconazole *vs.* the caspofungin group (*p* < 0.01); incidence of AKI in the voriconazole group was not significantly different than found in the other two groups. The infecting organism was a predictor of AKI and formulation with SBECD was not.

**Conclusions:**

Treatment of fungal infections in patients with compromised renal function with an SBECD-containing antifungal agent was not associated with AKI in clinical practice. Since the infecting organism was associated with AKI, decision on which antifungal to use should be determined by susceptibilities to the organism and not the incorporation of SBECD in the IV formulation.

## Background

The incidence of serious fungal infections has increased over the last two decades [1], coincident with increased prevalence of immunocompromised patients, including those treated for cancer, acquired immune deficiency syndrome (AIDS), and those treated with immunosuppressants in the setting of organ transplantation and autoimmune disease. Historically, yeasts were the most prevalent cause of fungal infections with *Candida albicans* being the most frequently identified. More recently, yeast infections have declined following the introduction and wide spread use of fluconazole and lipid-based amphotericin-B formulations. Further, there has been a shift in the frequency of isolates from *C. albicans* to non-albicans species, most notably *C. glabrata and C. parapsilosis*[2,3]. This decline in Candidal infections was associated with a rise in mould infections including aspergillosis [4]. More recently, the incidence of *Aspergillus* infections has been stable, [5,6]. However, a rising incidence of nosocomial infections by other filamentous fungi has been observed. These previously ultra-rare fungal diseases, such as *Scedosporium* spp., *Fusarium* spp., and *Zygomycetes* spp., are becoming more prevalent, particularly in immunocompromised patients in tertiary care cancer centers [7,8].

The increase in the availability of agents that are available to treat fungal infections allows individual agents to be selected based on the sensitivity of the organism and the potential toxicity profile of the antifungal agent. The high incidence of renal failure associated with the administration of polyenes has led to sensitivity regarding adverse renal effects of antifungal agents. Nephrotoxicity associated with the administration of first generation β-cyclodextrins [9] led to warnings and restrictions that have made it difficult to study the nephrotoxicity of later generation cyclodextrins. SBECD is a second-generation cyclodextrin chemically-engineered not to accumulate in renal epithelial cells and thus avoid acute kidney injury. Despite limited evidence that SBECD has not been associated with renal impairment [10,11], restrictions in its use in patients with estimated creatinine clearances (Cl_cr_) less than or equal to 50 mL/min have been documented [12].

Our study is designed to test the hypothesis that treatment of a known or suspected fungal infection that is serious enough to warrant the use of an intravenous agent in patients with impaired renal function at the start of therapy with an SBECD-containing antifungal is associated with a higher frequency of renal dysfunction than treatment with a non-SBECD containing antifungal. To date, there have been no comprehensive evaluations incorporating large population sizes of clinical populations in the literature. We found in our study that the inclusion of SBECD was not associated with drug-associated kidney injury in acutely-ill patients with systemic fungal infections.

## Results

A total of 128 patients with baseline Cl_cr_ < 50 mL/min and meeting all inclusion and exclusion criteria were analyzed from the aforementioned database. Fifty-five patients received caspofungin, 54 patients received fluconazole, and 19 patients received IV voriconazole. The groups were well matched with regard to age, race, comorbidities, and the concomitant administration of a known nephrotoxic agent (Table 1). There were significantly more women in the fluconazole group and this difference could not be attributed to treatment of a mucosal infection. Further, a disproportionate number of patients with diagnosed aspergillosis were treated with voriconazole. Approximately three-quarters of the voriconazole patients were treated with a concomitant nephrotoxic agent, as compared with 56% and 50% of those treated with caspofungin and fluconazole, respectively. A total of 21 (16%) patients received multiple concomitant nephrotoxic agents: 5 (26%) voriconazole patients (all receiving 2 concomitant nephrotoxic agents); 10 (18%) caspofungin patients (70%, 10%, and 20% receiving 2, 3, and 4 nephrotoxic agents, respectively); and 6 (11%) fluconazole patients (50% of patients each receiving 2 or 3 concomitant nephrotoxic agents).

**Table 1:** Patient characteristics

**Parameter**	**Voriconazole**	**Caspofungin**	**Fluconazole**	** p-value for overall difference**
**n (%)**	**19 (14.8)**	**55 (43.0)**	**54 (42.2)**	
Age, Mean (SD)	61.7 (13.9)	63.9 (13.9)	63.7 (14.7)	0.83
Male, n (%)	10 (52.6)	34 (61.8)	20 (37.0)	0.03
White, n (%)	17 (89.5)	43 (78.2)	49 (90.7)	0.63
Length of treatment (days)				
Mean (SD)	8.1 (6.3)	8.6 (5.3)	9.8 (9.8)	0.62
Median (IQR)	6.0 (4–12)	7.0 (5–11)	8.0 (5–11)	0.53
Min, Max	2, 29	2, 27	2, 74	NS
Comorbidities: Diabetes mellitus, hypertension or nephropathy, n (%)	11 (57.9)	46 (83.6)	43 (79.6)	0.06
Underlying condition, n (%)				0.001
Cancer	12 (63)*	7 (13)	13 (24)	
HIV	0	0	1	
ICU	15 (79)	31 (56)	26 (48)	
Immunosuppression**	12 (63)	3 (5)	12 (22)	
More than 1 underlying condition	12 (63)	9 (16)	16 (30)	
Concomitant nephrotoxic agent, n (%)	14 (73.7)	31 (56.4)	27 (50.0)	0.20
Underlying fungal disease, n (%)*				0.002
*Candida* spp.	5 (55.6)	43 (93.5)	39 (92.9)	
*Aspergillus* spp.	4 (44.4)	3 (6.5)	3 (7.1)	
Indication for antifungal therapy, n (%) ^†^				NS
Prophylaxis	10 (53)	8 (15)	5 (9)	
Empiric	0	1 (2)	5 (9)	
Presumed or Confirmed	9 (47)	46 (84)	42 (78)	

*10 patients with AML were treated with voriconazole prophylaxis.

**Chemotherapy, transplant rejection prevention or treatment, ≥ 1 mg/kg prednisone.

^†^Cases were included only when an Infectious Diseases consultant determined that the isolate represented an infection rather than colonization.

The underlying conditions are also listed in Table 1. More than half of the patients were being treated in the intensive care unit. Seventy-five percent of all patients had presumed or confirmed fungal disease, as determined by an Infectious Diseases consultant (9 [47%], 46 [84%], and 42 [78%] patients administered voriconazole, caspofungin, and fluconazole, respectively, NS; Table 1).

Baseline renal functional indices are listed in Table 2. Significant differences in baseline S_cr_ and blood urea nitrogen (BUN) levels by treatment group were present; the caspofungin group had higher levels than both of the other treatment groups. However, the differences in baseline estimated Cl_cr_ values were not significant among the groups. Cl_cr_ values, as calculated by the Cockcroft-Gault method or the MDRD equation, were not significantly different; hence, all comparisons were made using the Cockcroft-Gault method.

**Table 2:** Renal function at the start of therapy

**Parameter**	**Voriconazole**	**Caspofungin**	**Fluconazole**	**p-value for overall difference**
Baseline S_cr_ (mg/dL)	2.1 (0.9)	2.8 (1.5)	2.2 (1.3)	0.04
Baseline BUN (mg/dL)	33.3 (31.9)	56.5 (39.8)	48.7 (28.7)	0.04
Baseline Cl_cr_ (mL/min)				
Cockcroft-Gault method	33.9 (9.0)	28.2 (12.0)	30.4 (10.3)	0.14
MDRD^1^ calculation	33.3 (14.0)	28.4 (17.1)	32.1 (15.5)	0.36

^1^Modification of Diet in Renal Disease.

**Figure 1:**
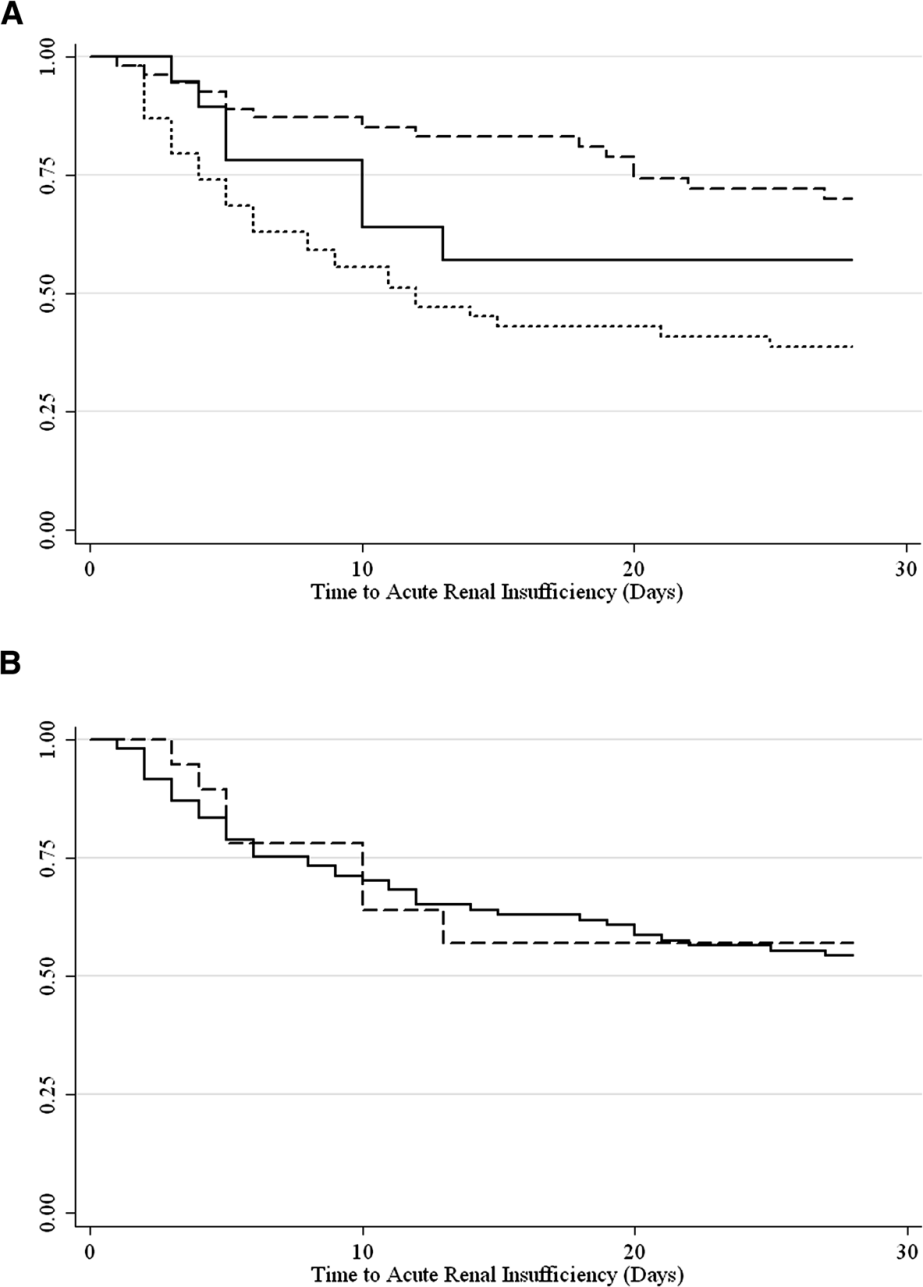
**A: Kaplan-Meier plot of patients who meet the RIFLE criteria by treatment with intravenous voriconazole (**^**____**^**), caspofungin (**^**_ _ _ _**^**), and fluconazole (**^**. . . . .**^**). B**: Kaplan-Meier plot of patients who meet the RIFLE criteria by formulations containing SBECD (^____^) and those with formulations which do not contain SBECD (^_ _ _ _^).

The changes in renal function as a function of time and antifungal agent or formulations containing SBECD and not containing SBECD are presented in Figures 1A and 1B, respectively. Non-SBECD containing agents had significantly lower nadir Cl_cr_ values than those observed with the SBECD containing antifungal, as reflected by change in the RIFLE criteria as well as absolute values (Table 3). Similarly, there were significant differences of maximal increase from baseline S_cr_ levels among the groups with the non-SBECD containing agents having greater increases than the SBECD containing agent. There were no significant differences among the groups in the incidence of renal dysfunction at the time of antifungal agent completion of therapy, as stratified by baseline renal function of Cl_cr_ < 30 mL/min or Cl_cr_ 30 – 50 mL/min (EOT; Table 3).

**Table 3:** Differences in renal function during therapy

**Parameter**	**Voriconazole**			**Caspofungin**			**Fluconazole**		
Baseline Cl_cr_ (mL/min)	<30	30–50	*p*-value	<30	30–50	*p*-value	<30	30–50	*p*-value
n (%)	6 (31.6)	13 (68.4)	--	33 (60.0)	22 (40.0)	--	27 (50.0)	27 (50.0)	--
Maximum ↑ S_cr_, mg/dL (SD)	0.68 (1.01)	0.62 (0.54)	0.88	1.17 (1.9)	0.15 (0.78)	0.02	2.6 (2.7)	2.0 (2.0)	0.33
Lowest Cl_cr_ during treatment, mL/min (SD)	16.8 (3.7)	31.7 (7.3)	0.0002	15.8 (6.0)	34.6 (7.7)	<0.0001	13.9 (5.1)	24.3(8.2)	<0.0001
Cl_cr_ at EOT, mL/min (SD)	28.7 (18.1)	50.3(27.8)	0.11	46.4(43.1)	59.5(28.4)	0.21	30.6(19.2)	49.0(22.6)	0.0025
% Change Cl_cr_ (Lowest-SOT)/SOT (SD)	−24.6 (12.6)	−19.6 (13.4)	0.45	−19.4(17.7)	−15.6(15.1)	0.41	−35.3(18.4)	−37.5(20.7)	0.68
Renal dysfunction, n (%)	3 (50.0)	4 (30.8)	0.42	12 (36.4)	3 (13.6)	0.06	17 (63.0)	15 (55.6)	0.58

S_cr_: serum creatinine; Cl_cr_: creatinine clearance; EOT: end of therapy, SOT: start of therapy.

The differences in 28-day mortality among the three treatment groups were not statistically significant. Mortality of patients in the voriconazole, caspofungin, and fluconazole treatment groups was 42.1%, 29.1%, and 25.9% respectively (*p* = 0.44).

Stepwise logistic regression analyses were performed using the following 7 independent variables: age, race, comorbidities, concomitant treatment with a nephrotoxic agent, the presence or absence of SBECD, and site of infection which was recoded into 3 groups (abdominal + lung, systemic, and urine + wound). The coefficient for infecting organism had a Wald statistic equal to 3.90 and a *p*-value of 0.048. Thus, this model identified the infecting organism as the only significant predictor of renal dysfunction by the RIFLE criteria.

## Discussion

This study found no evidence of renal toxicity that was attributable to the solubilizing agent, sulfobutylether-β-cyclodextrin (SBECD), in patients with impaired renal function (Cl_cr_ < 50 mL/min) who were treated with an intravenous antifungal agent in clinical practice. The maximal increase in S_cr_ levels was less and calculated nadir Cl_cr_ was higher for the SBECD-containing voriconazole group than those of fluconazole and caspofungin groups that did not contain SBECD. Importantly, SBECD was not associated with increased renal dysfunction during or at the end of therapy.

Voriconazole, a second-generation triazole, is a steric inhibitor of fungal cytochrome P450-dependent 14α-sterol demethylase, which is essential for the conversion of lanosterol to ergosterol required for building the cell membranes of fungal organisms [13]. Like other triazoles, voriconazole has activity for this enzyme for both yeast and moulds, whereas fluconazole, the backbone structure of voriconazole, is only active against yeast. The early development plan for voriconazole incorporated the medical need for an intravenous formulation [written communication, Chris Hitchcock, PhD, Pfizer Inc]. Since voriconazole has a low aqueous solubility (0.7 mg/mL) [14], its intravenous formulation includes SBECD as a solubilizing agent.

Naturally occurring cyclodextrins, notably α- and β-cyclodextrins, are reabsorbed and concentrated in the renal tubule, resulting in acute kidney injury at clinically-translatable doses in animal models [9]. SBECD is a rationally-designed cyclodextrin which does not undergo significant tubular reabsorption, thus it was formulated to prevent injury to renal tubular cells. Animal studies with SBECD have not shown discrete changes in renal morphology at doses used clinically [11]; moreover, SBECD is not nephrotoxic in these models when given at dose equivalents of naturally occurring cyclodextrins that are associated with nephrotoxicity and/or hepatotoxicity. However, at the time of FDA approval, a lack of information regarding the safety of SBECD in patients with impaired renal function led to warnings against the use of SBECD in patients with Cl_cr_ < 50 mL/min for products including intravenous voriconazole [12]. This study is important to clinicians because it provides evidence that SBECD-containing medications can be used to treat patients with serious fungal infections including those with impaired renal function without increased risk of nephrotoxicity.

Our main finding that treatment of a serious fungal infection for patients with impaired renal function with SBECD-containing voriconazole is not associated with worse renal outcomes than treatment with a non-SBECD containing alternative antifungal is consistent with recent smaller studies. A retrospective analysis of the *Candida* Phase 3 trial database for nephrotoxic effects of intravenous voriconazole containing SBECD did not detect renal toxicity from voriconazole in contrast to the renal dysfunction associated with amphotericin B [10,15]. Despite accumulation of SBECD in the plasma of patients with compromised renal function [16-18], its lack of reabsorption in renal epithelial cells appears to prevent deleterious effects on renal function in this and other clinical reports [19-21].

Targeting high risk patients may have increased the ability of our study of modest size to detect differences among agents with regard to renal toxicity. The association of fluconazole with worse renal function during therapy is consistent with its labeling: “In some patients, particularly those with serious underlying diseases such as AIDS and cancer, changes in renal and hematological function test results and hepatic abnormalities have been observed during treatment with fluconazole and comparative agents, but the clinical significance and relationship to treatment is uncertain [22].” Our findings do not identify fluconazole as a direct nephrotoxin, rather they imply that the underlying disease state of which fluconazole is treating influences renal function. This is consistent with our identification of the organism as a significant predictor of renal outcome. The association was driven by worse renal function among those with systemic fungal infections, many of whom were treated with fluconazole for sensitive isolates of *Candida albicans*. It is important to note that all cases of renal impairment were classified as mild-to-moderate according to the RIFLE criteria [23]. We did not find evidence for other possible mechanisms of renal toxicity such as obstructive hydronephrosis [24-31] or microangiopathy such as that which occurs with hemolytic uremic syndrome [32,33].

This study has important limitations that should be taken into account when interpreting the data. It is a nonrandomized observational study of clinical practice at an academic medical center with policies that mandate the involvement of infectious disease experts for cases involving the administration of intravenous antifungals for nearly all patients. This leads to differences among the groups that are due to non-random factors that may be important. On the other hand, this approach may be more informative about the effects in clinical practice because cases are not excluded. We attempted to review patient cases with compromised renal function treated with amphotericin B but, despite our large database, we could not find a sufficient number of patients meeting this criteria. Similarly, the number of patients with compromised renal function treated with intravenous voriconazole is low and likely related to labeling that raises concerns about renal toxicity in this group.

Mortality was higher in the voriconazole group compared with the other two groups, likely as a result of the severity of the underlying fungal disease. A greater percentage of patients in the voriconazole group was infected with *Aspergillus* spp. A higher mortality rate is expected in this group because infection with *Aspergillus* spp. has been associated with higher mortality rates compared to yeast infections. In addition, a greater percentage of patients in the voriconazole group were treated with voriconazole prophylactically as part of a treatment plan for poor prognosis malignancies including acute myelogenous leukemia (AML). Importantly, differences among the indications for antifungal treatment did not appear to alter the validity of the association of the infecting organism that was isolated with the development of renal dysfunction. Nonetheless, there was a limited number of *Candida* and *Aspergillus* isolates identified in this study such that associations between the subspecies of yeast or mould with more severe renal disease could not be definitive.

Another limitation of this study is the lack of dosing information or therapeutic drug monitoring. At the time of data collection, there was no harmonization between pharmacy and medical records. However, there are defined guidelines for the dosing of the three IV antifungals established by the Food and Drug Administration which were reviewed by a UMass clinical pharmacist. Antifungal agent levels were not measured as part of this study and are not recommended by current therapeutic guidelines. The effects of SBECD on renal function that we observed do not suggest that measuring drug levels to prevent renal toxicity would be justified.

## Conclusions

A main finding of this study is that the administration of SBECD in renally-compromised patients does not result in renal damage that is attributable to its inclusion in the formulation of voriconazole. The finding that the infecting organism is a significant predictor of renal outcomes in this high risk population suggests that the efficacy of the antifungal agent should be the dominant consideration when treating a serious fungal infection. Further studies are warranted to help us better understand the association between the underlying fungal infection and acute kidney injury.

## Methods

### Patients/samples

We analyzed deidentified data from a large database of hospitalized patients from the UMass Memorial Healthcare System using the University of Massachusetts Biorepository database. All adults with documented or clinically suspected fungal infections that were treated with an intravenous antifungal therapy, specifically, voriconazole, fluconazole, or caspofungin, with baseline estimated Cl_cr_ < 50 mL/min, and who were hospitalized between 1/1/2007 and 12/31/2010 were identified. Those eligible for analysis had been dosed for a minimum of 4 days of IV antifungal therapy and had serum creatinine (S_cr_) levels while on therapy and at the end of IV antifungal therapy (EOT; ± 3 days). Patients were excluded from analyses if they had unstable renal function at baseline (assessed as a 50% difference in S_cr_ or estimated Cl_cr_ on two measurements within 4 days prior to start of IV antifungal therapy). Patients undergoing chronic hemodialysis, hemofiltration, or other renal replacement therapies were excluded from study. All concomitant medications which could be considered nephrotoxic were evaluated in a blinded fashion (by DRL) and subsequently tabulated by treatment group (by VLW). The indication for treatment with an antifungal agent was classified as for prophylaxis, empiric treatment, or presumed/confirmed disease utilizing the EORTC guidelines [34].

S_cr_ levels below 0.7 mg/dL were rounded up to 0.7 mg/dL per standard protocol [35,36] Cl_cr_ was estimated by the Cockcroft-Gault equation:

$$\frac{Cl_{\mathrm{cr}}=\left(140-\mathrm{Age}\right)\times \mathrm{ABW}\times \left[0.85\;\mathrm{if}\;\mathrm{Female}\right]}{72\times S_{\mathrm{cr}}}$$

Age is in years, ABW is actual body weight in kilograms, and S_cr_ is measured in milligrams per deciliter [37-39]. Given the controversy in the literature regarding the best estimator of renal function [36], we also evaluated renal function by the Modification of Diet in Real Disease (MDRD) study equation [40]:

$$\begin{array}{l} Cl_{\mathrm{cr}}=\mathrm{eGFR}\\ \;=186\times {\left(S_{\mathrm{cr}}\right)}^{-1.154}\times {\left(\mathrm{AGE}\right)}^{-0.203}\\ \;\times \left(0.742\;\mathrm{if}\;\mathrm{Female}\right)\times \left(1.212\;\mathrm{if}\;\mathrm{Black}\right) \end{array}$$

This study was performed with the prior approval of the University of Massachusetts Human Subjects Committee under a waiver of the requirement for informed consent.

### Statistical analysis

Changes in S_cr_ levels and Cl_cr_ were evaluated during IV antifungal therapy. The highest S_cr_ and lowest Cl_cr_ while on therapy and at EOT among the groups were compared with baseline S_cr_ and Cl_cr_, respectively, by F-test. We compared dichotomous variables with the Fisher’s exact test, and other categorical variables with the χ^2^ test. Continuous variables were compared using the *F* test or Wilcoxon rank-sum test and differences among groups with the log-rank test. Distributions of length of treatment were skewed, so these data are shown as median (IQR), and other data are shown as mean (SD). We assessed development of renal dysfunction with RIFLE scores [23] by linear test of trend. 2-sided *p* values < 0.05 were considered statistically significant.

Stepwise logistic regression was conducted with a *p*-value to enter and stay in the model set for *p*≤0.20. The dependent variable in the model was the presence of AKI. Prespecified potential independent variables included age, gender, body mass index, antifungal therapy, infecting organism, presence or absence of SBECD, site of infection, Cl_cr_ at baseline, presence or absence of concomitant potentially nephrotoxic agents, and comorbidities. For all analyses except the regression model, *p* < 0.05 was considered significant. Analyses were conducted using SAS Software version 9.2 (Cary, North Carolina, USA).

## Abbreviations

ABW: Actual body weight; AIDS: Acquired immune deficiency syndrome; AKI: Acute kidney injury; AML: Acute myelogenous leukemia; BUN: Blood urea nitrogen; Cl_cr_: Creatinine clearance; EOT: End of therapy; IQR: Interquartile range; IV: Intravenous; MDRD: Modification of diet in renal disease; RIFLE criteria: Risk, injury, failure, loss of function, and end-stage renal disease; SBECD: Sulfobutylether-β-cyclodextrin; S_cr_: Serum creatinine; SD: Standard deviation; SOT: Start of therapy.

## Competing interests

DRL and VLW were employees of Pfizer Pharmaceuticals at the time of study. CML, TM, PR, and JM are employees of the University of Massachusetts with no financial competing interests. All analyses were performed with a database blinded to treatment allocation.

## Authors’ contributions

CML, VLW, and DRL conceived and developed the protocol, sought funding from Pfizer Pharmaceuticals, assessed the statistical outcomes, and drafted the manuscript. CML, TM, and PR collected the data and developed the blinded database. JM assessed quality assurance of the clinical data. VLW and CML analysed the database in a blinded fashion. All authors read and approved the final manuscript.

## Pre-publication history

The pre-publication history for this paper can be accessed here:

http://www.biomedcentral.com/1471-2334/13/14/prepub
